# The Role of Hemocytes in the Hawaiian Bobtail Squid, *Euprymna scolopes*: A Model Organism for Studying Beneficial Host–Microbe Interactions

**DOI:** 10.3389/fmicb.2016.02013

**Published:** 2017-01-06

**Authors:** Sarah J. McAnulty, Spencer V. Nyholm

**Affiliations:** Department of Molecular and Cell Biology, University of Connecticut, StorrsCT, USA

**Keywords:** symbiosis, hemocytes, *Euprymna scolopes*, *Vibrio fischeri*, innate immunity, invertebrate models

## Abstract

Most, if not all, animals engage in associations with bacterial symbionts. Understanding the mechanisms by which host immune systems and beneficial bacteria communicate is a fundamental question in the fields of immunology and symbiosis. The Hawaiian bobtail squid (*Euprymna scolopes*) engages in two known symbioses; a binary relationship with the light organ symbiont *Vibrio fischeri*, and a bacterial consortium within a specialized organ of the female reproductive system, the accessory nidamental gland (ANG). *E. scolopes* has a well-developed circulatory system that allows immune cells (hemocytes) to migrate into tissues, including the light organ and ANG. In the association with *V. fischeri*, hemocytes are thought to have a number of roles in the management of symbiosis, including the recognition of non-symbiotic bacteria and the contribution of chitin as a nutrient source for *V. fischeri*. Hemocytes are hypothesized to recognize bacteria through interactions between pattern recognition receptors and microbe-associated molecular patterns. Colonization by *V. fischeri* has been shown to affect the bacteria-binding behavior, gene expression, and proteome of hemocytes, indicating that the symbiont can modulate host immune function. In the ANG, hemocytes have also been observed interacting with the residing bacterial community. As a model host, *E. scolopes* offers a unique opportunity to study how the innate immune system interacts with both a binary and consortial symbiosis. This mini review will recapitulate what is known about the role of hemocytes in the light organ association and offer future directions for understanding how these immune cells interact with multiple types of symbioses.

## Introduction

Studies in a number of animal-microbe symbioses have revealed that bacteria are active in a wide range of biological processes (McFall-Ngai et al., 2013), including digestion, host defense, development, and even camouflage (Gil-Turnes et al., 1989; McFall-Ngai and Montgomery, 1990; Jones and Nishiguchi, 2004; Kau et al., 2011; Hooper et al., 2012; Fraune et al., 2015; Gromek et al., 2016). Interactions between hosts’ immune systems and the resident microbiota are often essential in the establishment and maintenance of beneficial associations (Stappenbeck et al., 2002; Nyholm and Graf, 2012; Buchon et al., 2013).

All animal immune systems must also strike a balance between promoting healthy microbiota and mounting a robust defense against pathogens. In order to maintain healthy associations, hosts must be able to distinguish between beneficial symbionts and unwanted microbial intruders. This recognition is often mediated by interactions between host pattern recognition receptors (PRRs) and microbe-associated molecular patterns (MAMPs). These MAMPs may include microbial components like peptidoglycan, lipopolysaccharide (LPS), outer membrane proteins, fimbriae, and bacterial flagellar proteins (Koropatnick et al., 2004; Fischer et al., 2006; Nyholm and Graf, 2012; Shen et al., 2012; Chu and Mazmanian, 2013).

Immune cells with phagocytic function, often referred to as hemocytes among invertebrates, are major components of the cellular innate immune system. Hemocytes are primarily implicated in defense against pathogens (Hillyer et al., 2003; Hillyer and Strand, 2014; Parsons and Foley, 2016), but also have metabolic functions in some species, including lipid storage and transport in mussels, oysters, snails, and insects (Allen and Conley, 1982; Pollero et al., 1985; Pollero and Garin, 1992; Adamo et al., 2008). In a number of beneficial associations, hemocytes have been found to both mediate and be influenced by the microbiota (Nyholm and McFall-Ngai, 1998; Silver et al., 2007; Nyholm et al., 2009; Rodrigues et al., 2010; Schmitz et al., 2012; Weiss et al., 2012; Schleicher et al., 2014). For example, in tse-tse flies, proper immune development, including immune gene expression and hemocyte proliferation, is dependent upon colonization by *Wigglesworthia* (Weiss et al., 2012). A similar effect is seen in pea aphids, wherein the presence of some secondary symbionts (e.g., *Hamiltonella defensa, Serratia symbiotica*, and *Regiella insecticola*) influences hemocyte abundance and the proportion of granulocytes in the hemocyte population (Schmitz et al., 2012). In the medicinal leech, the crop bacterium *Aeromonas veronii* has been shown to prevent phagocytosis by hemocytes through a type III secretion system (Silver et al., 2007).

**FIGURE 1:**
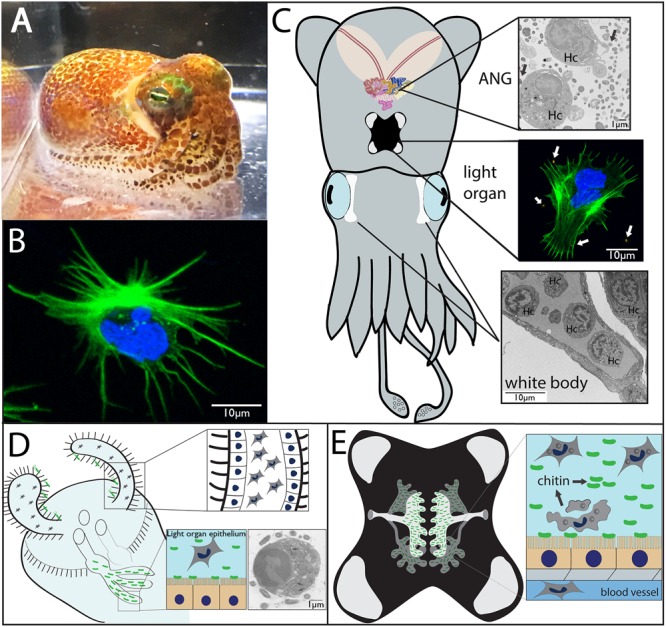
**Morphology and symbiotic organs of *Euprymna scolopes*. (A)** Hawaiian bobtail squid (*Euprymna scolopes*). **(B)** Hemocyte from *E. scolopes* as viewed by confocal microscopy and stained for actin (green) and DNA (blue). **(C)** Cartoon depicting the two main symbiotic organs, the light organ (middle) and the accessory nidamental gland [ANG, top, along with the white bodies (sites of hematopoiesis, bottom)]. Hemocytes (Hc) from the vasculature migrate into both the ANG (transmission electron micrograph) and light organ (confocal micrograph showing a hemocyte expelled from the light organ after venting) where they interact with bacteria (arrows). **(D)** Cartoon depicting juvenile light organ showing hemocytes that have trafficked into the sinuses of the ciliated appendages (inset, close up). Hemocytes may also migrate into the crypt spaces where they interact with *V. fischeri* and phagocytize bacteria (inset, TEM, internalized bacteria indicated by arrows; from Nyholm and McFall-Ngai, 1998). **(E)** Adult light organ showing the complex crypts spaces that house *V. fischeri*. The crypt spaces are connected directly with the environment via two pores on either side of the light organ. Although always present, more hemocytes migrate into the crypt spaces each night where a subset of these cells undergoes lysis, releasing chitin that serves as a nutritional source for the symbionts and promotes bioluminescence (inset).

Like many other decapod cephalopods, the Hawaiian bobtail squid, *Euprymna scolopes*, is thought to have only one morphological type of hemocyte that flows through a closed circulatory system along with the extracellular oxygen-binding protein hemocyanin (**Figures 1A,B**; Nyholm and McFall-Ngai, 1998; Kremer et al., 2014). In cephalopods, hemocytes develop in a hematopoietic organ, the white body, which is a highly vascularized 4-lobed organ located between the eyes and optic lobe (**Figure 1C**; Claes, 1996). In *E. scolopes*, mature hemocytes migrate from the white body into vasculature where they can then traffic through tissues including two prominent organs that house symbiotic bacteria; the light organ with the single symbiont *Vibrio fischeri* and the accessory nidamental gland, housing a bacterial consortium (**Figure 1**; ANG, a female reproductive organ).

## The Squid-Vibrio Association

For the past 30 years, the squid-vibrio symbiosis has been studied as a model association to investigate host–microbe interactions including the influence of beneficial bacteria on innate immunity and development (Nyholm and McFall-Ngai, 2004; McFall-Ngai, 2014). *E. scolopes* provides a tractable host for studying microbial interactions with the innate immune system due to both the simplified binary symbiosis found in the light organ that is home to the single bacterial symbiont, *Vibrio fischeri*, (**Figures 1C–E**) and the consortial symbiosis found in the female squid ANG (**Figure 1C**). The symbiosis with *V. fischeri* allows the nocturnal host to use light produced by the symbiont to mask itself from predators while hunting, in a behavior known as counterillumination (Jones and Nishiguchi, 2004). The juvenile organ is poised to recruit *V. fischeri* from the environment using pairs of ciliated epithelial appendages that extend from either side of the organ and entrain seawater containing *V. fischeri* and other bacteria (**Figure 1D**; McFall-Ngai and Ruby, 1991; Lee and Ruby, 1992, 1994; Nyholm and McFall-Ngai, 2004). Through a number of cellular, biomechanical, and biochemical mechanisms, successful colonization occurs when *V. fischeri* cells enter the pores, migrate through ciliated ducts, and make their way to the epithelium-lined crypts (Nyholm and McFall-Ngai, 2004; McFall-Ngai, 2014). *V. fischeri* is the only bacterium that is able to make the journey to the crypt spaces and successfully colonize the light organ. Morphogenesis of the light organ occurs after successful colonization, including apoptosis and regression of the ciliated appendages (McFall-Ngai and Ruby, 1991; Montgomery and McFall-Ngai, 1994; Foster and McFall-Ngai, 1998). These developmental events are triggered by MAMPs from *V. fischeri* including LPS and tracheal cytotoxin (TCT, a derivative of peptidoglycan), along with outer membrane vesicles (OMVs) (Foster et al., 2000; Koropatnick et al., 2004; Aschtgen et al., 2016).

## Hemocyte Interactions With *V. fischeri*

In addition to the biochemical and biophysical hurdles mentioned above, *V. fischeri* also encounters hemocytes in the light organ (**Figures 1C–E**; Nyholm and McFall-Ngai, 1998; Koropatnick et al., 2007). Upon initiation of the symbiosis, as few as 3–5 *V. fischeri* cells induce hemocyte trafficking into the ciliated appendages of the nascent organ via signaling by TCT and OMVs (Koropatnick et al., 2007; Altura et al., 2013; Aschtgen et al., 2016). Hemocytes have also been shown to internalize OMVs (Aschtgen et al., 2016) but how these vesicles and other MAMPs are detected by hemocytes is unclear. These cells do express a number of PRRs, including peptidoglycan recognition proteins (PGRPs), a Toll-like receptor (TLR), and galectins (see below; Collins et al., 2012a; Schleicher et al., 2014). The role of hemocytes in light organ development is not completely understood, but these cells are hypothesized to be involved in restructuring of the ciliated appendages during morphogenesis and after the initiation of symbiosis (Koropatnick et al., 2007). A previous study implicated that gelanolytic activity via a matrix metalloproteinase (MMP) may contribute to this process (Koropatnick et al., 2014) and other work has shown an increase in MMP expression in the light organs of colonized juveniles (Chun et al., 2008) and in hemocytes from symbiotic compared to antibiotic-treated (cured) adult hosts (Collins et al., 2012a; Schleicher et al., 2014). However, regression of the ciliated appendages may not be entirely dependent upon hemocytes, as squid colonized by *V. fischeri* in simulated microgravity showed a decrease in hemocyte trafficking but an accelerated restructuring of the ciliated appendages (Foster et al., 2013).

In symbiotic juvenile animals, there was a significant increase in hemocyte trafficking to the crypt spaces by 36 h post-colonization (Koropatnick et al., 2007). Another study also observed hemocytes with internalized bacteria in the light organ crypt spaces of colonized juvenile hosts (**Figure 1D**; Nyholm and McFall-Ngai, 1998). These observations led to the question of whether hemocytes can distinguish between symbiotic and non-symbiotic bacteria. Hemocytes are easy to isolate, especially in adult hosts through the cephalic blood vessel, and are amenable to *in vitro* studies (Collins and Nyholm, 2010), although no immortalized cell line from any cephalopod has been developed. Hemocytes isolated from the circulation of the host can survive for a number of days in culture, and live cell experiments that monitor binding of fluorescently labeled bacteria can easily be performed (Nyholm et al., 2009). An *in vitro* study using adult hosts showed hemocytes from colonized squid bound significantly fewer cells of *V. fischeri* and the closely related but non-symbiotic *Vibrio parahaemolyticus* than *Vibrio harveyi* or *Photobacterium leiognathi* (Nyholm et al., 2009). However, once the light organ was cured, hemocytes bound significantly more *V. fischeri*, while binding to all non-symbiotic bacteria remained unchanged. These findings suggest that hemocytes can distinguish between related bacteria (all strains tested were within the *Vibrionaceae*) and that colonization state influences the binding behavior of the hemocytes, specifically toward *V. fischeri*. These observations have led to investigations of the molecular mechanisms by which colonization alters the cellular immune response of the host (Collins et al., 2012a; Schleicher et al., 2014).

A combination of both symbiont and host factors are likely involved with mediating a change in hemocyte tolerance to *V. fischeri*. A *V. fischeri* mutant lacking the porin, outer membrane protein U (OmpU), bound to hemocytes significantly more than wild-type (WT) *V. fischeri* ES114 (Nyholm et al., 2009). WT *V. fischeri* may release immunomodulatory molecule(s) via this porin that mediate binding to hemocytes. However, because curing experiments lead to a global change in hemocyte binding response to *V. fischeri*, transmitted signal(s) from the symbiont likely influence the host systemically either through the circulatory system directly or via hemocytes that encounter *V. fischeri* and migrate from the light organ crypts to other organs in the body. Future studies will characterize whether colonization influences hematopoiesis in *E. scolopes* and whether *V. fischeri* transmits signals to developing hemocytes in the white body.

## Light Organ Colonization Influences Hemocyte Gene Expression and Protein Abundance

**FIGURE 2:**
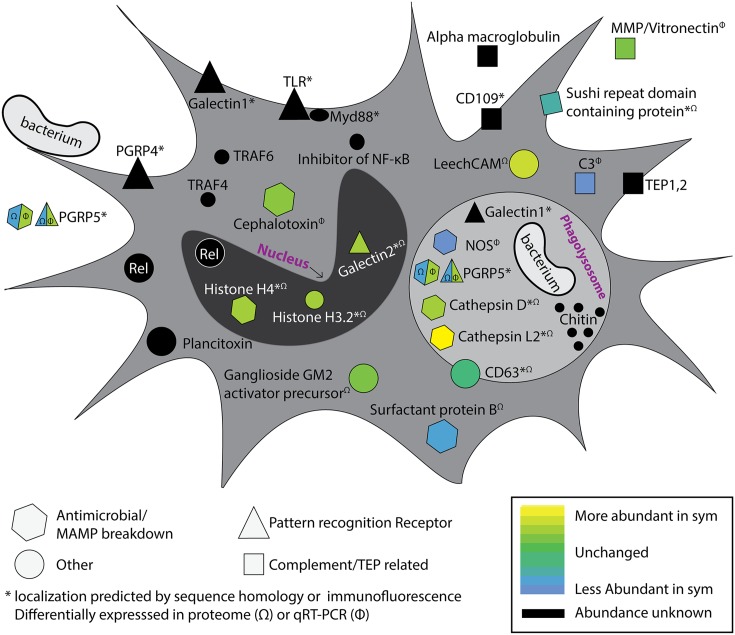
**Colonization by *Vibrio fischeri* influences the systemic hemocyte transcriptome and proteome.** Proteomics and transcriptomics on hemocytes from colonized and cured squid have revealed a number of immune-related genes and proteins that are differentially expressed/abundant between colonization states (Collins et al., 2012a; Schleicher et al., 2014). Multiple putative pattern recognition receptors (PRRs) were identified, along with predicted components of the complement cascade, antimicrobial enzymes, and NF-κB pathway. Differential expression is indicated by heat map (bottom right). Genes and proteins that were detected in the transcriptome of circulating hemocytes but were not observed to be differentially expressed are indicated in black. Proteins differentially abundant in proteomic analysis are indicated with (Ω) and transcripts differentially expressed by qRT-PCR indicated with (Φ). Protein localizations are based on protein homology or immunofluorescence; indicated with asterisk (^∗^). EsPGRP5 is a PRR with predicted amidase activity and showed opposite differential expression patterns at the transcript and protein levels (see text). Acronyms defined as follows: MMP, matrix metalloproteinase; TEP, thioester containing protein; CD109, cluster of differentiation 109; PGRP, peptidoglycan recognition protein; TLR, toll-like receptor, TRAF, TNF Receptor Associated Factor; Rel, Relish; NOS, nitric oxide synthase; LeechCAM, Leech cell adhesion molecule; CD63, cluster of differentiation 63; C3, Complement component 3. Modified from Collins et al. (2012a).

To understand how colonization state influences hemocyte gene and protein expression, two studies employed transcriptomics and proteomics to investigate circulating hemocytes in adult hosts (Collins et al., 2012a; Schleicher et al., 2014). Transcriptomics and proteomics of circulating hemocytes revealed a number of innate immune-related genes and proteins, some of which were differentially expressed between colonized and cured squid (**Figure 2**). These included a peptidoglycan recognition protein (*EsPGRP5*), complement component C3, nitric oxide synthase, a MMP, and cephalotoxin (Collins et al., 2012a). *EsPGRP5* was among the most abundant transcripts expressed in hemocytes and is predicted to have amidase activity, meaning it has the potential to degrade bacterial peptidoglycan. A previous study demonstrated that the amidase activity of another PGRP (*EsPGRP2*) likely plays a role in mediating interactions with *V. fischeri* in the light organ crypts and during the onset of the symbiosis (Troll et al., 2010). A quantitative proteomic analysis of hemocytes from colonized and cured animals identified 1,024 proteins, of which 37 proteins were differentially abundant between colonization states (Schleicher et al., 2014). Among these were proteins involved in cytoskeletal restructuring and cell adhesion (beta-tubulin, gelsolin, stathmin), cell stress (heat shock proteins), lysosomal proteins (cathepsins, ganglioside gm2 activator precursor), proteases, and other innate immune proteins (sushi von Willebrand factor type A). One of the most abundant proteins in sym hemocytes was a lysosomal cathepsin L2. Cathepsins are proteases and have been reported to be modulated by beneficial and pathogenic microorganisms (Nepal et al., 2006; Thomas et al., 2008; Nishikori et al., 2009; Schleicher et al., 2014). Protein abundance of PRRs also differed between colonization states, including a galectin (more abundant in sym), and *Es*PGRP5 (less abundant in sym). qRT-PCR showed that *EsPGRP5* gene expression was up-regulated in symbiotic hosts but this incongruence between the transcript and protein levels may indicate protein turnover and/or secretion of the protein (**Figure 2**; Collins et al., 2012a; Schleicher et al., 2014). Galectins are carbohydrate recognition proteins known to bind microbial surfaces (Vasta, 2009). In some systems they have been shown to influence phagocytosis and/or contribute to innate immune system specificity toward bacteria (Yu et al., 2007; Shi et al., 2014; Stowell et al., 2014). Members of the complement cascade in hemocytes and the light organ were also found to be differentially expressed depending on colonization state (Collins et al., 2012a; Schleicher et al., 2014; Yazzie et al., 2015). Combined, these “omics” data provide evidence that colonization by *V. fischeri* influences hemocyte gene expression and protein abundance. These findings, together with binding and phagocytosis assays (Nyholm et al., 2009), suggest colonization has a systemic effect on cellular innate immune function. The influence of colonization on hemocyte recognition of *V. fischeri* likely involves a complex signaling process between host and symbiont. Proteomic techniques have been valuable tools for identifying candidate effectors, and these hemocyte proteins and MAMPs are now being investigated experimentally for their roles in specific recognition. The particular signaling molecules (e.g., MAMPs) that may induce tolerance, and host proteins that are involved with specific bacterial binding, have yet to be characterized but this is an area of active investigation. Future studies will also investigate how colonization by *V. fischeri* influences hematopoiesis to determine whether tolerance of the symbiont is influenced by developmental changes in hemocyte progenitor cells. A number of innate immunity and hematopoiesis genes have been detected in the white body of the related squid, *Euprymna tasmanica*, but the role of this organ in symbiosis has not been studied (Salazar et al., 2015).

## Hemocytes as a Source of Chitin for the Light Organ Community

In addition to their role in distinguishing between symbiotic and non-symbiotic bacteria, hemocytes have recently been demonstrated to act as a nutrient source for *V. fischeri* in adult hosts (Schwartzman et al., 2015). Hemocytes have been observed in light organ crypt spaces (Nyholm and McFall-Ngai, 1998) where they increase in numbers during the evening when the symbiont population is greatest (Schwartzman et al., 2015). Like many other invertebrate hemocytes, blood cells from *E. scolopes* are rich in chitin (Heath-Heckman and McFall-Ngai, 2011). This chitin is localized in acidic compartments, presumably lysosomes, and delivered to the symbiotic population of the light organ at night after cell membrane blebbing and lysis of a subset of hemocytes (**Figure 1E**; Schwartzman et al., 2015). Diel transcriptional changes in both host and symbiont revealed an increase in expression of chitinases by the symbiont and chitotriosidase by the host at night (Wier et al., 2010). The lumen of the light organ becomes increasingly acidic during the evening due to catabolism of chitin by symbionts, eventually reaching a pH of 5.5 (Kremer et al., 2014). This acidic environment causes the oxygen-binding protein hemocyanin to release bound oxygen, which is required by *V. fischeri* for bioluminescence (Kremer et al., 2014). *V. fischeri* has been shown to chemotax toward host-derived chitobiose in the mucus secreted from the juvenile light organ during the initiation of the symbiosis, further underlining the importance of chitin in the association (Mandel et al., 2012). Hemocytes therefore act as sentinels, contributing to specificity in the light organ and as a source of chitin substrate for the symbionts, the catabolism of which also promotes bioluminescence, the main function of the association.

## *E. scolopes* as a Model for Binary and Consortial Symbioses

The nature of communication between hosts and their bacterial partners is complex and any experimental simplification is welcome, which makes the binary symbiosis between *E. scolopes* and *V. fischeri* such a powerful model for the investigation of host–microbe interactions. The host also offers a unique opportunity as a model organism because in addition to the light organ symbiosis, squid also host the bacterial community of the ANG (**Figure 1C**; Collins et al., 2012b). This gland is composed of many epithelium-lined tubules, containing a bacterial consortium dominated by members of the *Alphaproteobacteria* and *Verrucomicrobia* (Collins et al., 2012b). The bacteria are deposited into the egg jelly coats of squid where they are hypothesized to play a protective role during development (Collins et al., 2012b, 2015; Gromek et al., 2016). Like the light organ, the ANG is highly vascularized and hemocytes have been observed within the lumen of the tubules and in direct contact with bacteria (**Figure 1C**; Collins et al., 2012b). These observations raise two interesting lines of inquiry for future research. First, since only female squid have an ANG, are there sex-based differences in the innate immune response to different bacterial taxa? Sex-based differences in immune response have been observed in sea urchins, flies, birds, mice, and humans (reviewed in Klein and Flanagan, 2016). In a number of systems, including the scorpion fly, sea urchin, and wall lizard, immune cells from female animals show increased phagocytosis of foreign particles (Mondal and Rai, 1999; Kurtz et al., 2000; Arizza et al., 2013). Future studies may also reveal differences between male and female hemocyte responses to bacteria in *E. scolopes*. Second, the ANG and light organ symbioses offer the opportunity to ask how the innate immune system interacts with both a binary and a consortial symbiosis in the same host. Since both organs are highly vascularized, there may be multiple levels of hemocyte-mediated specificity given that interactions with hemocytes occur with various types of bacteria across different host tissues. A previous study showed that two host galaxins (*esgal1* and *esgal2*) were differentially expressed between the light organ and ANG. One of these galaxins (EsGal1) also had antimicrobial activity against marine Gram-positive bacteria and influenced the growth of *V. fischeri* (Heath-Heckman et al., 2014). Efforts are currently underway to characterize the systemic immune response of *E. scolopes* to colonization of each organ and to understand whether hemocyte tolerance to ANG bacteria occurs similarly to what has been described for *V. fischeri*.

## Conclusion

The squid-vibrio association offers a tractable system to study interactions between beneficial bacteria and the innate immune system without the additional complexity of antibody-based adaptive immunity common to the vertebrates. Previous studies show that *V. fischeri* can influence hemocyte dynamics in multiple ways. A number of pressing questions involving hemocytes in the squid-vibrio system still need to be addressed. Transcriptomics and proteomics have revealed potential PRRs that may be important in recognizing *V. fischeri* and other bacteria. The degree to which hemocytes influence the early interactions of the squid-vibrio association is also unclear. For example, given that only 3–5 founder cells are sufficient to initiate colonization (Altura et al., 2013), is binding and phagocytosis of *V. fischeri* further suppressed at hatching? Understanding how colonization state influences hemocyte recognition during the first 96 h of the association will also be critical in identifying any symbiosis-specific changes in hemocyte recognition of bacteria and mechanisms of immune tolerance to *V. fischeri*. Advances in *E. scolopes* husbandry (Hanlon et al., 1997; Koch et al., 2014) are also opening up opportunities to ask long-term developmental questions in the association that should shed light on how symbiosis influences the maturation of the innate immune response. Future hemocyte-based studies would also greatly benefit from the development of new tools in the host. A complete genome for *E. scolopes* is forthcoming (Albertin et al., 2012) and will assist with the identification of other immune-related genes. Targeted proteomics and the development of CRISPR-Cas technologies may also prove useful in expanding the experimental repertoire in this association. Finally, like many animals, *E. scolopes* must negotiate multiple microbial associations that are found in different organs and tissues. Further understanding of the ANG symbiosis along with our extensive knowledge of the light organ association may reveal conserved mechanisms by which the cellular innate immune system interacts with multiple types of microbes in different host environments.

## Author Contributions

All authors listed, have made substantial, direct and intellectual contribution to the work, and approved it for publication.

## Conflict of Interest Statement

The authors declare that the research was conducted in the absence of any commercial or financial relationships that could be construed as a potential conflict of interest.
